# BAP1 acts as a tumor suppressor in intrahepatic cholangiocarcinoma by modulating the ERK1/2 and JNK/c-Jun pathways

**DOI:** 10.1038/s41419-018-1087-7

**Published:** 2018-10-10

**Authors:** Xu-Xiao Chen, Yue Yin, Jian-Wen Cheng, Ao Huang, Bo Hu, Xin Zhang, Yun-Fan Sun, Jian Wang, Yu-Peng Wang, Yuan Ji, Shuang-Jian Qiu, Jia Fan, Jian Zhou, Xin-Rong Yang

**Affiliations:** 1 Department of Liver Surgery, Liver Cancer Institute, Zhongshan Hospital, Fudan University, 200032 Shanghai, China; 2 Key Laboratory of Carcinogenesis and Cancer Invasion, Ministry of Education, 200032 Shanghai, China; 3 Department of Laboratory Medicine, Zhongshan Hospital, Fudan University, 200032 Shanghai, China; 4 Department of Pathology, Zhongshan Hospital, Fudan University, 200032 Shanghai, China

## Abstract

Current therapeutic options for intrahepatic cholangiocarcinoma (ICC) are very limited, which is largely attributed to poor understanding of molecular pathogenesis of ICC. Breast cancer type 1 susceptibility protein-associated protein-1 (BAP1) has been reported to be a broad-spectrum tumor suppressor in many tumor types, yet its role in ICC remains unknown. The aim of this study was to investigate the clinical implications and biological function of BAP1 in ICC. Our results showed that the messenger RNA and protein levels of BAP1 were significantly downregulated in ICC versus paired non-tumor tissues. Overexpression of wild-type but not mutant BAP1 significantly suppressed ICC cell proliferation, cell cycle progression, and invasion in vitro, as well as tumor progression in vivo. Conversely, knockdown of BAP1 yielded opposing effects. Mechanistically, BAP1 functioned as a tumor suppressor in ICC by inhibiting the extracellular signal-regulated kinase 1/2 and c-Jun N-terminal kinase/c-Jun pathways, and this function was abolished by inactivating mutations. Clinically, low BAP1 expression was positively correlated with aggressive tumor characteristics, such as larger tumor size, presence of lymphatic metastasis, and advanced tumor node metastasis stage. Survival analysis revealed that low BAP1 expression was significantly and independently associated with poor overall survival and relapse-free survival after curative surgery. In conclusion, BAP1 is a putative tumor suppressor of ICC, and may serve as a valuable prognostic biomarker as well as potential therapeutic target for ICC.

## Background

Intrahepatic cholangiocarcinoma (ICC), arising from the malignant transformation of intrahepatic cholangiocytes, is the second most common primary hepatic malignancy^1–3^. As one of the most aggressive tumors, the incidence and mortality of ICC have been rapidly increasing worldwide, with geographic variation^4,5^. Surgical resection remains the mainstay of potentially curative therapy for patients with ICC, but the resectability rate is quite low because of the high frequency of metastases^6,7^. Even worse, no effective chemotherapies or molecular target therapies are available for ICC, which is mainly attributed to poor understanding of the molecular pathogenesis of this malignancy^8–10^. Therefore, a better understanding of the molecular mechanisms associated with ICC progression would benefit the development of new effective treatment modalities.

The ubiquitin–proteasome system (UPS) is an essential and highly regulated system in charge of 80–90% protein degradation and turnover, which is central to keeping intracellular protein homeostasis and regulating cellular function^11^. Many key proteins regulated by UPS are involved in tumor onset and progression, and mutations in UPS genes are implicated in various types of cancer^12–14^. Similar to protein phosphorylation, protein ubiquitination is a highly reversible process, and it can be reversed by a class of isopeptidases known as deubiquitinating enzymes (DUBs), which are involved in numerous biological processes, including transcriptional regulation, cell growth and differentiation, and oncogenesis^15,16^.

Breast cancer type 1 susceptibility protein (BRCA1)-associated protein-1 (BAP1) was originally identified as a novel DUB interacting with the RING finger domain of BRCA1^17^. It is a member of the ubiquitin carboxy-terminal hydrolase (UCH) subfamily of DUBs, and plays critical roles in key cellular processes including transcription, cell cycle regulation, cell differentiation, cell death, and DNA damage response^13,18,19^. BAP1 has been considered a true tumor suppressor and appears to follow a classic Knudson two-hit paradigm^20,21^. Germline or somatic mutations and deletions of BAP1 have been identified in various tumor types, and downregulation or inactivation of BAP1 could accelerate tumor onset, invasion, recurrence, and metastases^13,22–27^. Meanwhile, genetic evidence from mouse models carrying heterozygous germline BAP1 mutations showed that BAP1 was a bona fide tumor suppressor and mutant BAP1 mouse models exhibited a high incidence of neoplasms, including ovarian sex cord stromal tumors, lung carcinomas, and breast carcinomas, and so on^28^.

Recently, a relative high mutation frequency of BAP1 was identified in ICC by several exome sequencing projects^29,30^. Because of the implied significance of BAP1, we were compelled to investigate the clinical significance and biological function of BAP1 in ICC. In this study, we found that BAP1 was significantly downregulated in ICC, and its decreased expression correlated with poor overall survival (OS) and relapse-free survival (RFS) after curative surgery. Furthermore, results of functional assays indicated that BAP1 controlled ICC cell proliferation, cell cycle progression, and invasion in vitro, as well as tumor progression in vivo, by modulating the extracellular signal-regulated kinase 1/2 (ERK1/2) and c-Jun N-terminal kinase (JNK)/c-Jun pathways. Therefore, we proposed that BAP1 is a putative tumor suppressor in ICC, and may serve as a valuable prognostic biomarker as well as a potential therapeutic target in ICC.

## Results

### BAP1 is downregulated in human ICC and correlates with lymphatic metastasis

**Fig. 1:**
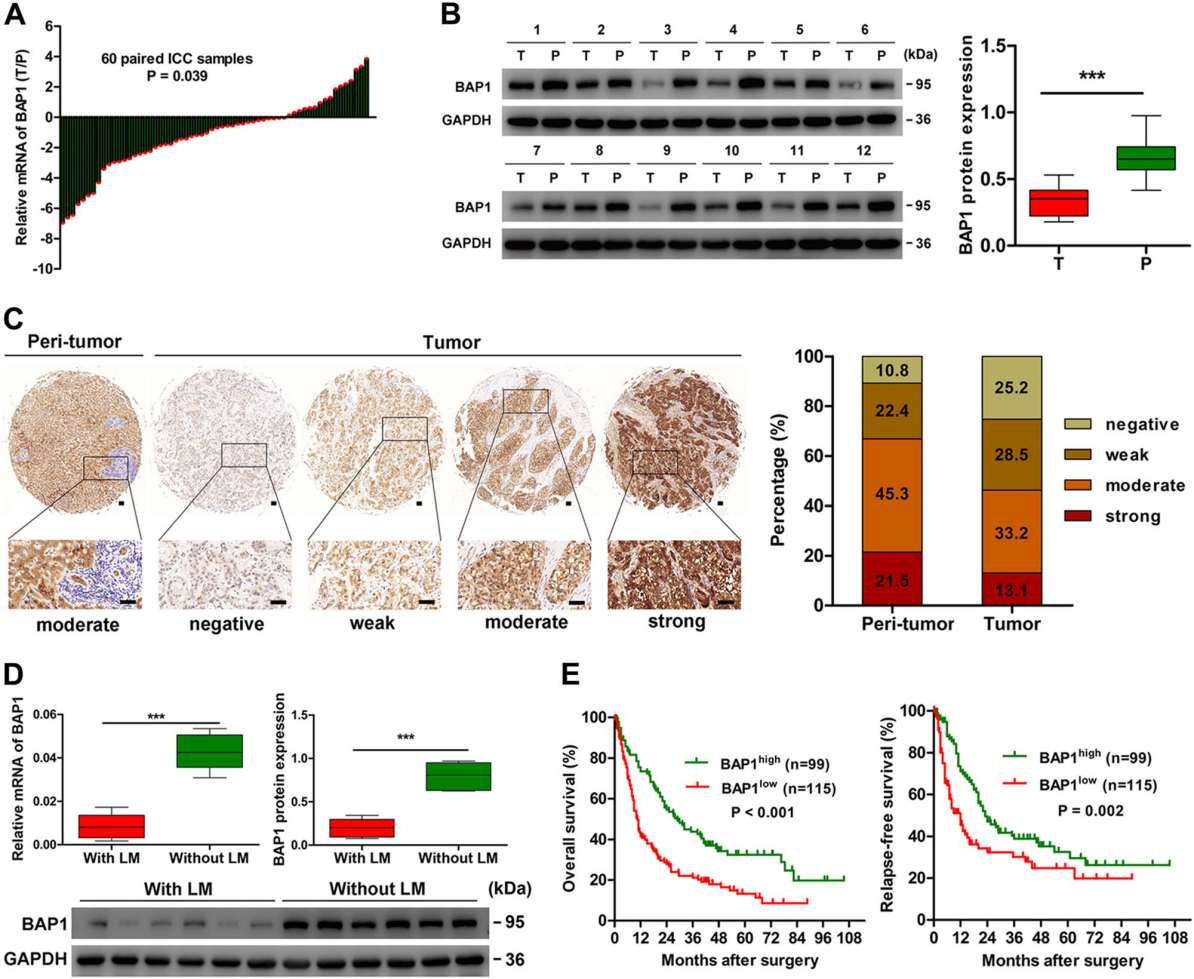
BAP1 is downregulated in human ICC and correlates with lymphatic metastasis. **a** The mRNA expression of BAP1 in 60 paired ICC tumor and adjacent non-tumor tissues. **b** The protein expression of BAP1 in 12 paired ICC tumor (T) and adjacent non-tumor tissues (P). Densitometry analysis for BAP1 was expressed relative to the loading control, GAPDH. **c** Representative immunostaining images of BAP1 in ICC and adjacent non-tumor tissues. Left: adjacent non-tumor tissues. Right: different staining intensities in ICC. Bar graph showed the statistics for the staining intensity of BAP1 in tissue microarrays containing 214 ICC patients. Scale bars = 50 μm. **d** The mRNA expression and protein expression of BAP1 in ICC with lymphatic metastasis and without lymphatic metastasis. Densitometry analysis for BAP1 was expressed relative to the loading control, GAPDH. **e** Kaplan–Meier curves for overall survival and relapse-free survival of ICC patients according to the expression of BAP1. All bar graphs depicted quantification of triplicate results with mean ± SD. ***P < 0.001

To explore the potential role of BAP1 in ICC, we first evaluated messenger RNA (mRNA) expression of BAP1 in 60 paired ICC samples and matched adjacent non-tumor liver tissues. The results showed that BAP1 mRNA expression was downregulated in 73.3% (44/60) of ICC tissues, relative to the adjacent non-tumor liver tissues (*P* = 0.039) (Fig. 1a). Western blot assays in 12 paired ICC tumor and adjacent non-tumor liver tissues showed similar results (Fig. 1b). Then, tissue microarray (TMA) assay was performed to detect the expression of BAP1 in ICC. Immunohistochemical data indicated that BAP1 was scored as a negative or weak expression in 53.7% (115/214) of tumor tissues, as compared with only 33.2% (71/214) in corresponding adjacent normal intrahepatic biliary tissues (Fig. 1c).

Compared to other tumor types, lymphatic metastasis is more prevalent in ICC and is an important predictive factor for poor prognosis^31^. Therefore, we analyzed another 12 independent ICC samples with (*n* = 6) or without lymphatic metastasis (*n* = 6), and found that BAP1 mRNA and protein expression were even lower in ICC with lymphatic metastasis than in those without lymphatic metastasis (Fig. 1d).

### Low BAP1 expression correlates with aggressive clinicopathological characteristics and poor prognosis after surgery in ICC

For the whole population of the 214 ICC patients, the 1-year, 3-year, 5-year, and 7-year OS and RFS rates were 58.4% and 57.8%, 31.7% and 33.4%, 22.1% and 27.8%, and 13.1% and 22.3%, respectively. To investigate the clinical implications of BAP1 in ICC, all the 214 ICC patients were dichotomized as BAP1^low^ (scored as negative or weak, *n* = 115) and BAP1^high^ (scored as moderate or strong, *n* = 99), according to the immunohistochemical data. Low BAP1 expression was significantly correlated with high serum CA19-9 level (*P* = 0.001) and aggressive tumor characteristics, such as larger tumor size (*P* = 0.010), presence of lymphatic metastasis (*P* < 0.001), and advanced tumor node metastasis (TNM) stage (*P* = 0.005) (Table 1). Low BAP1 expression was significantly associated with poor OS and RFS after curative surgery (Fig. 1e). Median OS and RFS in patients in the BAP1^low^ group were significantly shorter than patients in the BAP1^high^ group (OS, 10.5 versus 29.0 months, *P* < 0.001; RFS, 12.0 versus 23.0 months, *P* = 0.002). Of note, multivariate analysis, using Cox proportional hazards regression model adopting all significant variables in univariate analyses, confirmed that low BAP1 expression was an independent prognostic factor for OS (hazard ratio (HR) = 1.67, 95% confidence interval (CI): 1.20–2.32, *P* = 0.002) and RFS (HR = 1.52, 95% CI: 1.04–2.22, *P* = 0.031) (Table 2). Collectively, these data clearly indicated that low BAP1 expression was a valuable predicting factor for dismal prognosis after curative surgery in ICC patients.

**Table 1:** Correlation between BAP1 expression and clinicopathologic characteristics in 214 ICC patients

Clinicopathological indexes	BAP1		
	Low	High	*P* value*
Age (year)			
≤50	47	40	0.945
>50	68	59	
Sex			
Female	51	28	**0.015**
Male	64	71	
HBsAg			
Negative	66	46	0.111
Positive	49	53	
AFP (ng/ml)			
≤20	89	73	0.534
>20	26	26	
CA19-9 (U/ml)			
<37	42	59	**0.001**
≥37	73	40	
ALT (U/l)			
≤75	96	86	0.488
>75	19	13	
Liver cirrhosis			
No	104	84	0.212
Yes	11	15	
Tumor size (cm)			
≤5	37	49	**0.010**
>5	78	50	
Tumor number			
Single	106	87	0.292
Multiple	9	12	
Tumor encapsulation			
Complete	19	25	0.115
None	96	74	
Lymphatic metastasis			
No	79	88	**0.000**
No	36	11	
Vascular invasion			
No	91	84	0.280
Yes	24	15	
Tumor differentiation			
I–II	77	69	0.668
III–IV	38	30	
TNM stage			
I	44	57	**0.005**
II–III	71	42	

*AFP* α-fetoprotein, *CA19-9* carbohydrate antigen 19-9, *ALT* alanine aminotransferase

* *χ*^2^ tests for all analysis

*P* < 0.05, which indicate significantly difference

**Table 2:** Univariate and multivariate analyses of prognostic factors in 214 ICC patients

Variables	OS		RFS	
	HR (95% CI)	*P* value	HR (95% CI)	*P* value
*Univariate analysis*				
Age (year) (>50 versus ≤50)	1.361 (0.989–1.874)	0.059	1.363 (0.940–1.978)	0.103
Sex (male versus female)	1.193 (0.863–1.650)	0.285	1.403 (0.955–2.061)	0.085
HBsAg (positive versus negative)	0.751 (0.550–1.025)	0.071	0.957 (0.668–1.373)	0.813
AFP (ng/ml) (>20 versus ≤20)	0.862 (0.591–1.258)	0.442	0.786 (0.503–1.229)	0.291
CA19-9 (U/ml) (≥37 versus <37)	1.545 (1.130–2.112)	**0.006**	1.280 (0.891–1.838)	0.182
ALT (U/L) (>75 versus ≤75)	1.397 (0.917–2.128)	0.120	1.592 (0.993–2.553)	0.053
Liver cirrhosis (yes versus no)	0.979 (0.618–1.549)	0.926	1.125 (0.664–1.907)	0.661
Tumor size (cm) (>5 versus ≤5)	1.593 (1.154–2.199)	**0.005**	1.601 (1.102–2.325)	**0.013**
Tumor number (multiple versus single)	0.882 (0.500–1.557)	0.665	0.753 (0.381–1.486)	0.413
Tumor encapsulation (none versus complete)	1.940 (1.264–2.977)	**0.002**	2.414 (1.440–4.047)	**0.001**
Lymphatic metastasis (yes versus no)	2.531 (1.783–3.593)	**0.000**	1.894 (1.227–2.924)	**0.004**
Vascular invasion (yes versus no)	1.436 (0.977–2.109)	0.065	2.017 (1.318–3.086)	**0.001**
Tumor differentiation (III–IV versus I–II)	1.270 (0.912–1.770)	0.157	1.243 (0.844–1.830)	0.271
TNM stage (II + III versus I)	1.581 (1.158–2.160)	**0.004**	0.935 (0.650–1.345)	0.718
BAP1 (low versus high)	1.987 (1.445–2.730)	**0.000**	1.774 (1.230–2.557)	**0.002**
*Multivariate analysis*				
CA19-9 (U/ml) (≥37 versus <37)	NA	NA	NA	NA
Tumor size (cm) (>5 versus ≤5)	1.426 (1.030–1.973)	**0.032**	1.510 (1.034–2.207)	**0.033**
Tumor encapsulation (none versus complete)	1.728 (1.120–2.666)	**0.013**	2.173 (1.287–3.669)	**0.004**
Lymphatic metastasis (yes versus no)	1.978 (1.378–2.840)	**0.000**	1.595 (1.010–2.519)	**0.045**
Vascular invasion (yes versus no)	NA	NA	2.019 (1.305–3.125)	**0.002**
TNM stage (II + III versus I)	NA	NA	NA	NA
BAP1 (low versus high)	1.670 (1.201–2.322)	**0.002**	1.519 (1.039–2.222)	**0.031**

Cox proportional hazards regression model. Variables for multivariate analyses were adopted for their prognostic significance by univariate analysis (*P*<0.05), and these variables were assessed for prognostic significance by univariate analysis with forward stepwise selection (forward, likelihood ratio)

*HR* hazard ratio, *95% CI* 95% confidence interval, *AFP* α-fetoprotein, *CA19-9* carbohydrate antigen 19-9, *ALT* alanine aminotransferase, *NA* not applicable

*P* values are all < 0.05, which indicate significantly difference

### Downregulation of BAP1 promotes proliferation, cell cycle progression, and invasion of ICC in vitro

**Fig. 2:**
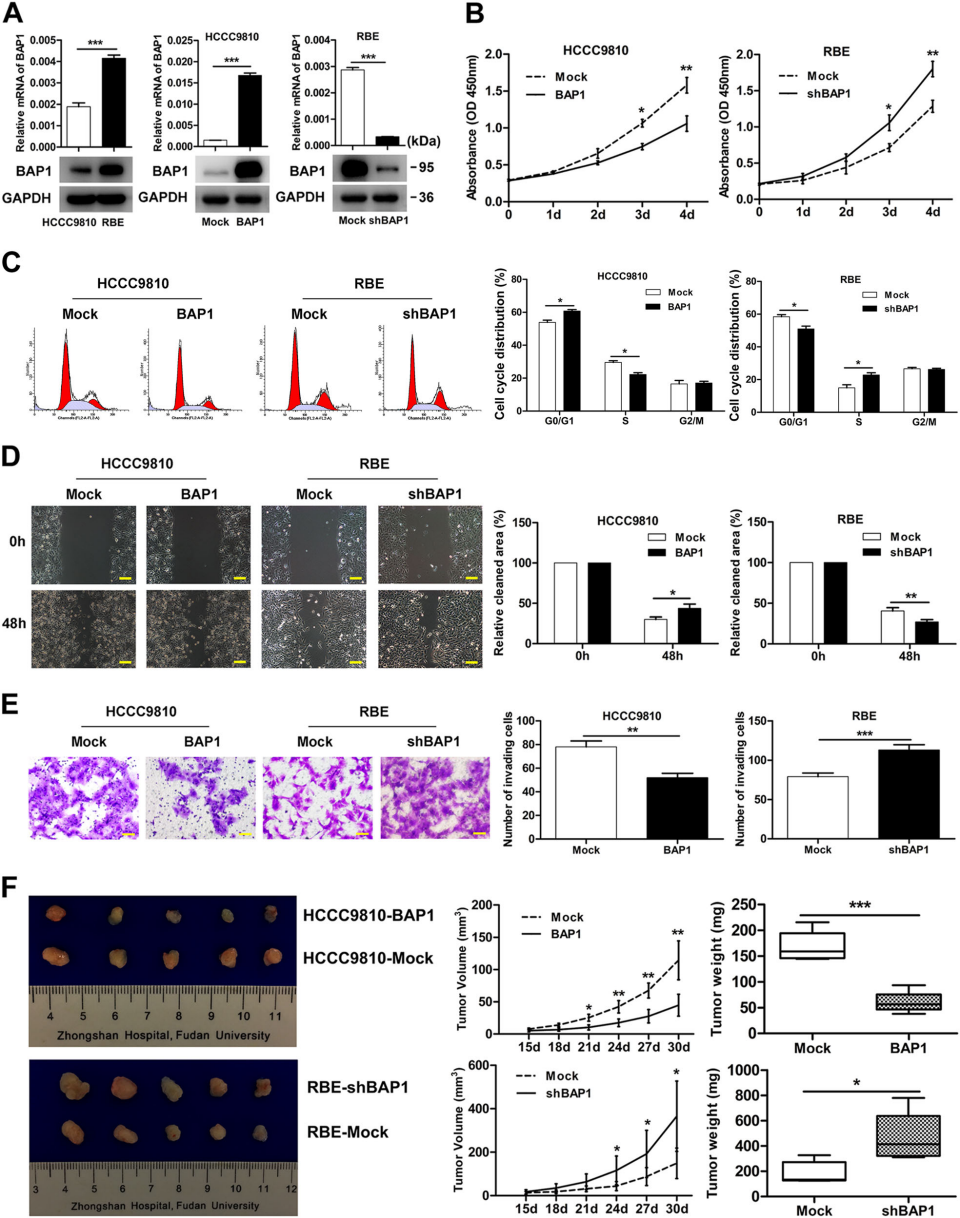
Downregulation of BAP1 promotes proliferation, cell cycle progression, and invasion of ICC in vitro and ICC progression in vivo. **a** The mRNA expression and protein expression of BAP1 in ICC cell lines (HCCC9810, RBE, HCCC9810-Mock, HCCC9810-BAP1, RBE-Mock, and RBE-shBAP1) were shown. **b** Effects of BAP1 overexpression and downregulation on proliferation using 3-(4,5-dimethylthiazol-2-yl)-2,5-diphenyltetrazolium bromide assay. **c** Effects of BAP1 overexpression and downregulation on cell cycle progression using flow cytometry after propidium iodide staining. Representative images were shown. **d** Effects of BAP1 overexpression and downregulation on migration using scratch wound healing assay. Representative images were shown. Scale bars = 200 μm. **e** Effects of BAP1 overexpression and downregulation on invasion using Matrigel invasion assay. Representative images were shown. Scale bars = 100 μm. **f** Effects of BAP1 overexpression and downregulation on the growth of in vivo subcutaneous xenograft tumors. Tumor volume and weight of xenografts derived from HCCC9810-BAP1 cells were significantly reduced as compared with those of tumors derived from HCCC9810-Mock cells (*n* = 5); tumor volume and weight of xenografts derived from RBE-shBAP1 cells were markedly increased as compared with those of tumors derived from RBE-Mock cells (*n* = 5). All bar graphs depicted quantification of triplicate results with mean ± SD. *P < 0.05, **P < 0.01, and ***P < 0.001

The clinical implications of BAP1 in ICC compelled us to explore its exact biological function. HCCC9810, a BAP1^low^ ICC cell line, was transfected with lentiviral vector encoding wild-type BAP1 to generate an ICC cell line with stable overexpression of BAP1 (HCCC9810-BAP1) and compared with its control (HCCC9810-Mock). RBE, a BAP1^high^ ICC cell line, was transfected with lentiviral vector encoding short hairpin BAP1 (shBAP1) to establish an ICC cell line with stable downregulation of BAP1 (RBE-shBAP1) and compared with its control (RBE-Mock) (Fig. 2a). BAP1 overexpression and downregulation efficiency were confirmed using real-time quantitative reverse transcription PCR (qRT-PCR), western blot (Fig. 2a), and immunofluorescence (Supplementary Figure S1).

First, in the proliferation analysis using the 3-(4,5-dimethylthiazol-2-yl)-2,5-diphenyltetrazolium bromide assay, overexpression of BAP1 in HCCC9810 cells led to significant inhibition of cell proliferation. In contrast, proliferation of RBE-shBAP1 cells was significantly enhanced compared to its control (Fig. 2b). Further cell cycle analysis revealed that overexpression of BAP1 in HCCC9810 cells significantly reduced cells in the S phase and arrested cells in the G0/G1 phase, which suggested that overexpression of BAP1 resulted in decreased G1-phase to S-phase cell cycle progression. Conversely, markedly increased G1-phase to S-phase cell cycle progression was detected in RBE cells after downregulation of BAP1 (Fig. 2c). Moreover, both scratching assay and Matrigel invasion assay indicated that overexpression of BAP1 in HCCC9810 cells significantly reduced cell migratory and invasion capacities, while the motility and invasion potential of RBE-shBAP1 cells were evidently enhanced compared to RBE-Mock cells (Fig. 2d, e). Taken together, these data revealed that downregulation of BAP1 promotes proliferation, G1 to S cell cycle progression, and invasion of ICC in vitro.

### **Downregulation of BAP1 promotes ICC progression** in vivo

Subsequently, mouse subcutaneous xenograft models were used to examine the effect of BAP1 on ICC progression in vivo. Tumor growth curves showed that tumors derived from HCCC9810-Mock and RBE-shBAP1 cells grew evidently faster than those from HCCC9810-BAP1 and RBE-Mock cells over the same period, respectively (Fig. 2f). Likewise, tumor volume of xenografts derived from HCCC9810-Mock and RBE-shBAP1 cells were 114.25 ± 30.02 and 365.45 ± 161.84 mm^3^, respectively, markedly larger than the volume of tumors derived from HCCC9810-BAP1 and RBE-Mock cells (44.65 ± 16.99 and 148.86 ± 70.07 mm^3^, *P* = 0.002 and *P* = 0.025, respectively). Similarly, tumor weight of xenografts derived from HCCC9810-BAP1 and RBE-Mock cells were significantly lighter than those of tumors derived from HCCC9810-Mock and RBE-shBAP1 cells (Fig. 2f). Together, these data suggested that downregulation of BAP1 promoted ICC progression in vivo.

### BAP1 regulates ICC cell proliferation, cell cycle progression, and invasion via inhibiting ERK1/2 and JNK/c-Jun signaling pathways

**Fig. 3:**
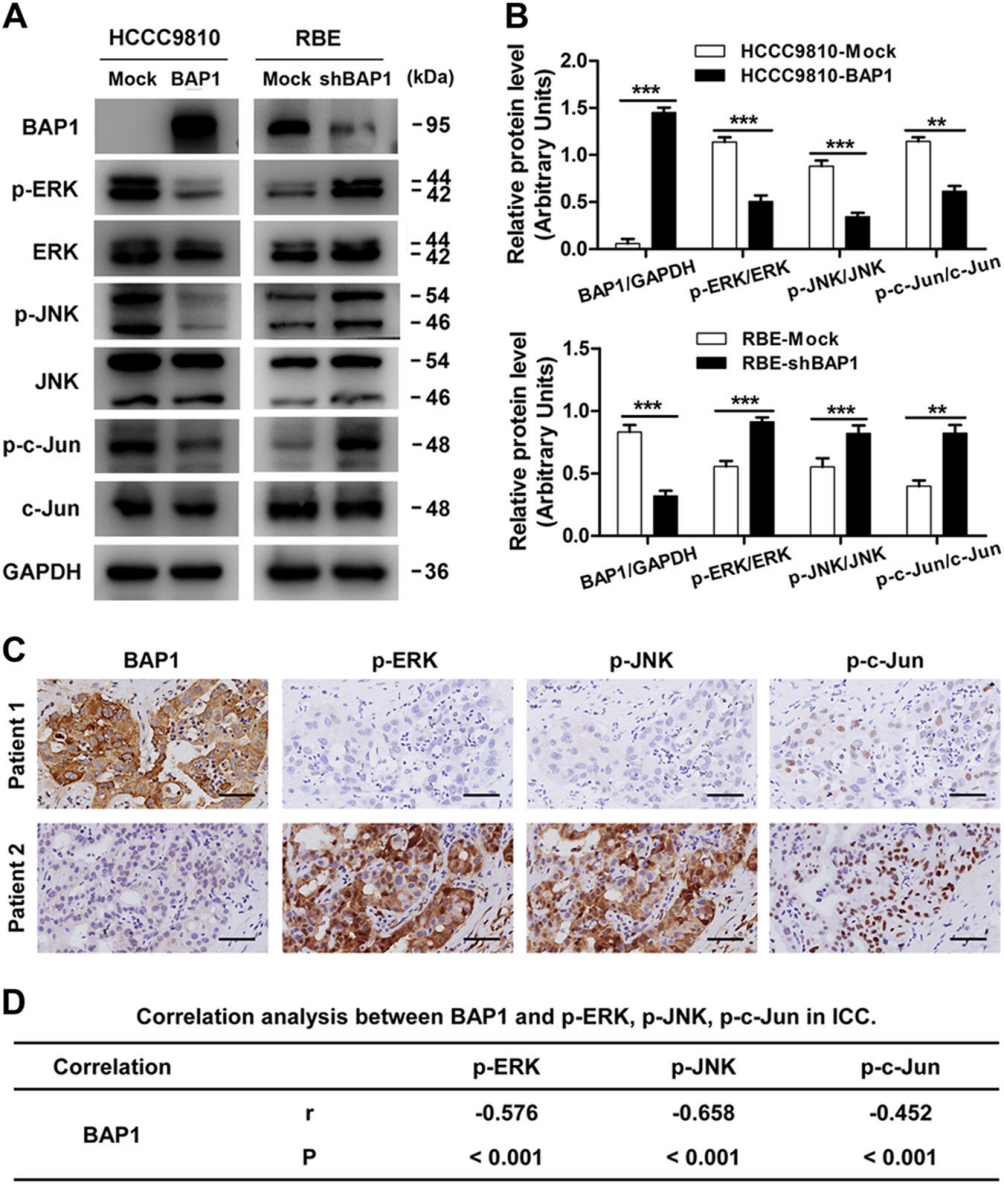
BAP1 inhibits ERK1/2 and JNK/c-Jun signaling pathways in ICC. **a** Protein levels of total and phosphorylation forms of ERK1/2, JNK, and c-Jun were compared in indicated cells. GAPDH was used as loading control. **b** Densitometry analysis was performed on three experiments representative of **a** and expressed relative to GAPDH or the corresponding total protein as the internal control. **c** Representative immunostaining images of BAP1 and phosphorylation forms of ERK1/2, JNK, and c-Jun in ICC samples. Scale bars = 50 μm. **d** Statistics of the correlation between BAP1 and p-ERK, p-JNK, and p-c-Jun in ICC. All bar graphs depicted quantification of triplicate results with mean ± SD. **P < 0.01 and ***P < 0.001

**Fig. 4:**
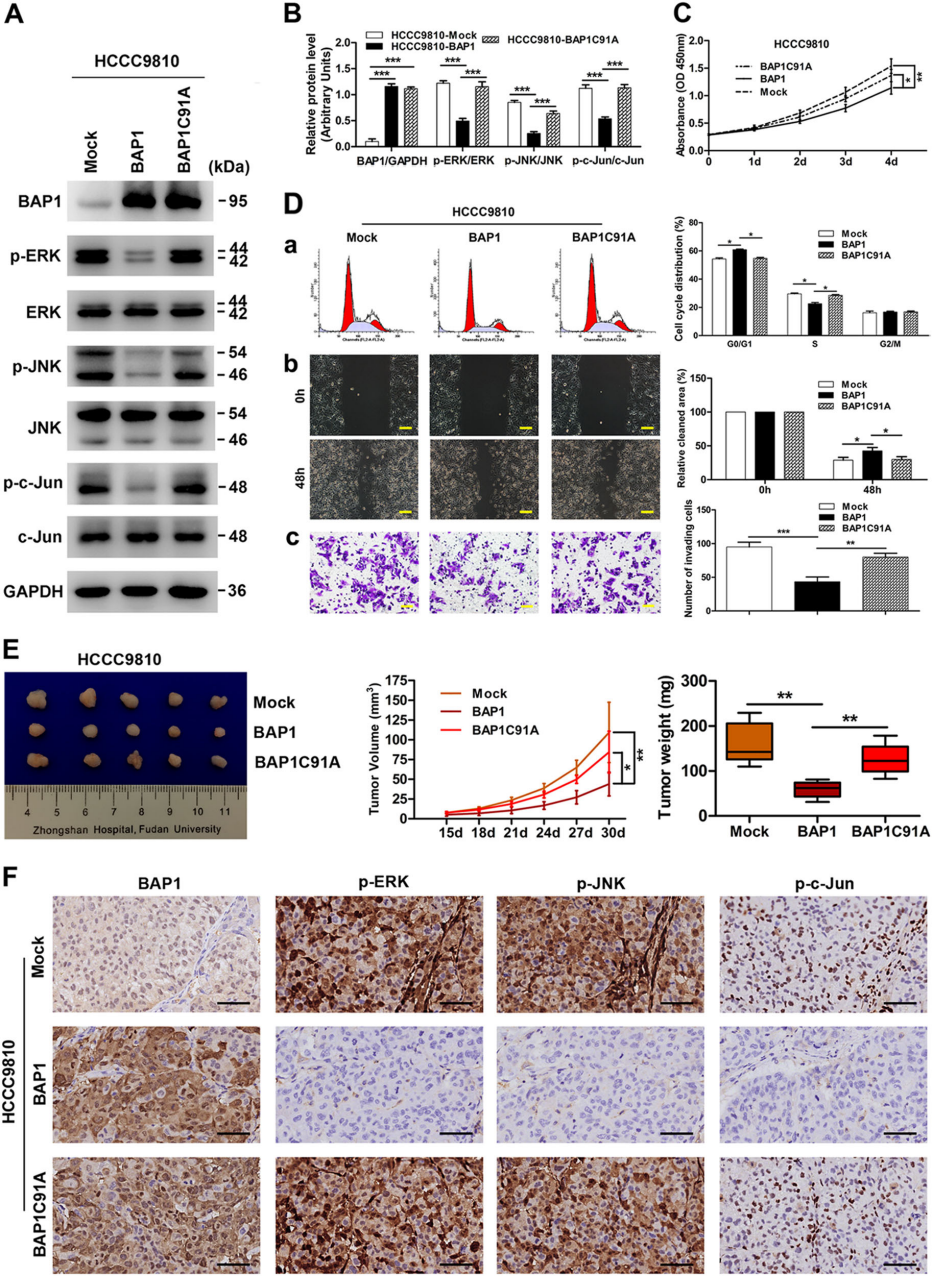
BAP1 regulates ICC cell proliferation, cell cycle progression, and invasion via inhibiting ERK1/2 and JNK/c-Jun signaling pathways. **a** Protein levels of total and phosphorylation forms of ERK1/2, JNK, and c-Jun were compared in indicated cells. GAPDH was used as a loading control. **b** Densitometry analysis was performed on three experiments representative of **a** and expressed relative to GAPDH or the corresponding total protein as the internal control. **c** Effects of the transfection of wild-type and inactive mutant BAP1 on cell proliferation. **d** Effects of the transfection of wild-type and inactive mutant BAP1 on cell cycle progression (a), migration (b), and invasion (c). Representative images were shown. Scale bars = 200 μm (b) and 100 μm (c). **e** Effects of the transfection of wild-type and inactive mutant BAP1 on the growth of in vivo subcutaneous xenograft tumors. **f** Representative images from tumor sample serial sections stained with BAP1, p-ERK, p-JNK, and p-c-Jun for each group. Scale bars = 50 μm. All bar graphs depicted quantification of triplicate results with mean ± SD. *P < 0.05, **P < 0.01, and ***P < 0.001

ERK1/2 and JNK/c-Jun signaling pathways play vital roles in many physiological processes including cell proliferation, differentiation, survival, and death, and persistent activation of ERK1/2 and JNK/c-Jun signaling has been observed in a high percentage of cancers, including ICC^32,33^. Moreover, UCHs have been reported to exert their functions in oncogenesis through modulating mitogen-activated protein kinase (MAPK) signaling pathways^34^. Therefore, we wondered whether BAP1, as an important member of UCHs, functioned in ICC through regulating ERK1/2 and JNK/c-Jun signaling pathways. As expected, western blot analysis showed that the phosphorylation of ERK1/2, JNK, and c-Jun were significantly downregulated in HCCC9810-BAP1 and RBE-Mock cells as compared to HCCC9810-Mock and RBE-shBAP1 cells, respectively (Fig. 3a, b). We further investigated the phosphorylation level of ERK1/2, JNK, and c-Jun in TMAs containing 214 ICC patients. Consistently, the immunohistochemical results also showed a significant negative correlation of expression of BAP1 with the phosphorylation level of ERK1/2, JNK, and c-Jun (Fig. 3c, d). Then, we transfected one inactive mutant BAP1C91A plasmid into HCCC9810 cells, producing HCCC9810-BAP1C91A cells. Western blot assays showed that the phosphorylation of ERK1/2, JNK, and c-Jun were significantly downregulated in HCCC9810-BAP1 cells but not in HCCC9810-BAP1C91A cells, as compared to HCCC9810-Mock cells (Fig. 4a, b). Likewise, cell proliferation, G1-phase to S-phase cell cycle progression, and invasion of HCCC9810 cells were significantly inhibited by transfection of wild-type BAP1 but not inactive mutant BAP1C91A (Fig. 4c, d). Consistently, the in vivo study also suggested that overexpression of wild-type BAP1 but not inactive mutant BAP1C91A evidently inhibited ICC progression, and the immunohistochemical results also confirmed that the inhibition of ERK1/2 and JNK/c-Jun signaling pathways were mediated by wild-type BAP1 but not by inactive mutant BAP1C91A (Fig. 4e, f). These results indicated that BAP1 suppressed ICC cell proliferation, cell cycle progression, and invasion via inhibiting ERK1/2 and JNK/c-Jun signaling pathways.

**Fig. 5:**
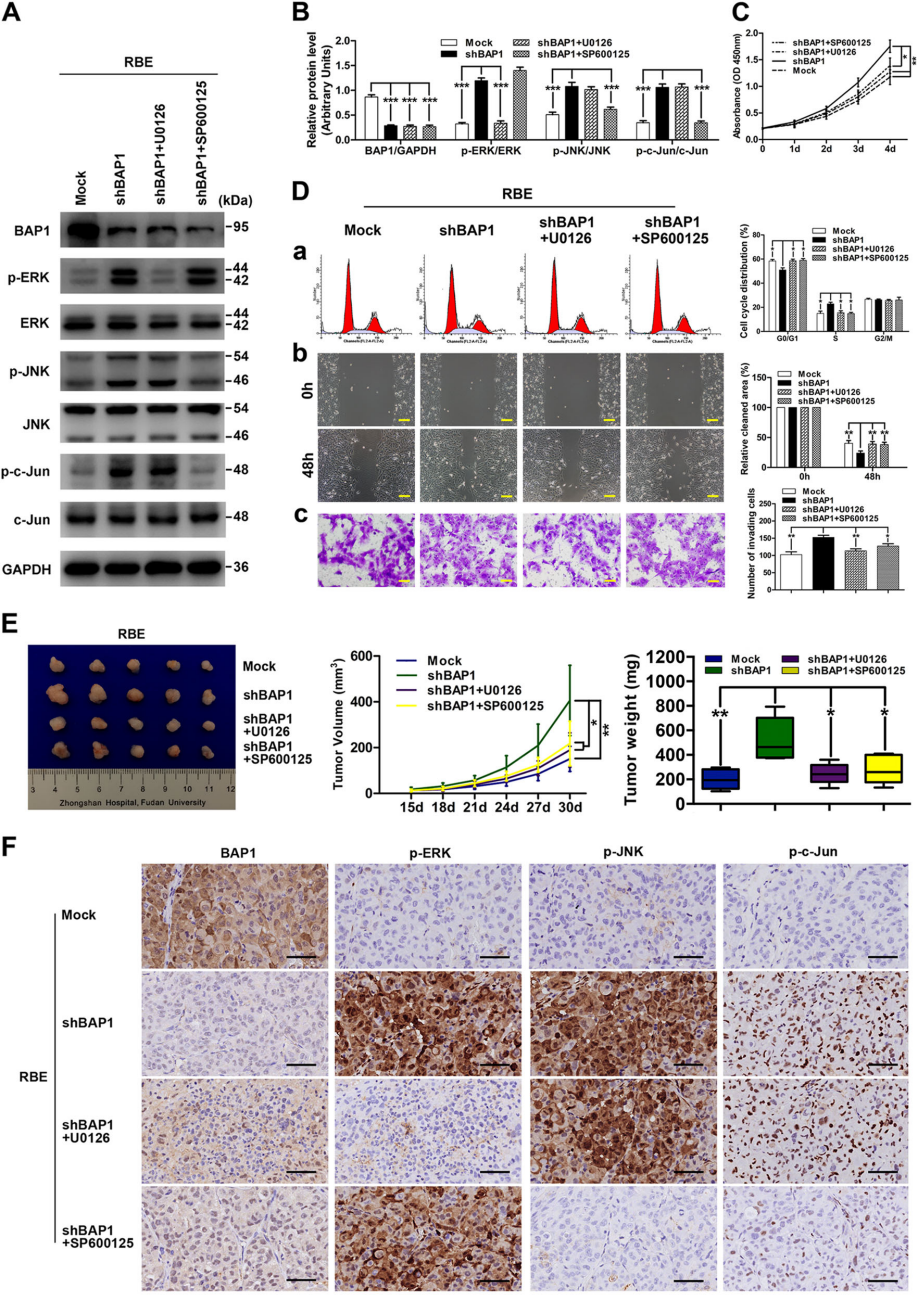
Hyperactivity of ERK1/2 and JNK/c-Jun signaling induced by BAP1 downregulation was necessary for ICC cell proliferation, cell cycle progression, and invasion. **a** Protein levels of total and phosphorylation forms of ERK1/2, JNK, and c-Jun were compared in indicated cells. GAPDH was used as loading control. **b** Densitometry analysis was performed on three experiments representative of **a** and expressed relative to GAPDH or the corresponding total protein as the internal control. **c** Effects of inhibitors of the ERK1/2 pathway (U0126) and JNK/c-Jun pathway (SP600125) on cell proliferation. **d** Effects of inhibitors of the ERK1/2 pathway (U0126) and JNK/c-Jun pathway (SP600125) on cell cycle progression (a), migration (b), and invasion (c). Representative images were shown. Scale bars = 200 μm (b) 100 μm (c). **e** Effects of inhibitors of the ERK1/2 pathway (U0126) and JNK/c-Jun pathway (SP600125) on the growth of in vivo subcutaneous xenograft tumors. **f** Representative images from tumor sample serial sections stained with BAP1, p-ERK, p-JNK, and p-c-Jun for each group. Scale bars = 50 μm. All bar graphs depicted quantification of triplicate results with mean ± SD. *P < 0.05, **P < 0.01, and ***P < 0.001

To further validate these findings, we used inhibitors of the ERK1/2 pathway (U0126) and JNK/c-Jun pathway (SP600125) in RBE-shBAP1 cells. Western blot analysis was performed to assess the effect of U0126 and SP600125 on the phosphorylation of ERK1/2, JNK, and c-Jun. As expected, both the inhibitors successfully suppressed the phosphorylation of the corresponding proteins (Fig. 5a, b). More importantly, cell proliferation, G1-phase to S-phase cell cycle progression, and invasion of RBE-shBAP1 cells were significantly inhibited after treatment with either U0126 or SP600125 (Fig. 5c, d). Consistently, the in vivo study also suggested that inhibition of ERK1/2 or JNK/c-Jun signaling pathways using U0126 or SP600125 abrogated the pro-ICC effect induced by BAP1 downregulation (Fig. 5e, f). These data clearly suggested that hyperactivity of ERK1/2 and JNK/c-Jun signaling induced by BAP1 downregulation is necessary for ICC cell proliferation, cell cycle progression, and invasion. All of these results further confirmed that BAP1 suppressed ICC cell proliferation, cell cycle progression, and invasion via inhibiting ERK1/2 and JNK/c-Jun signaling pathways.

## Discussion

In the present study, we investigated the clinical significance, biological function, and the underlying mechanisms of BAP1 in ICC pathogenesis and progression. Our data demonstrated that BAP1 is a potential tumor suppressor in ICC. More importantly, our results also confirmed that BAP1 deficiency increased the phosphorylation and activity of ERK1/2 and JNK/c-Jun signaling to promote ICC progression. Our findings of the functional characterization of BAP1 in ICC may provide a novel potential therapeutic target for future drug development.

In this study, our data showed that BAP1 expression was significantly downregulated in ICC. Consistently, results from previous studies in other types of cancer, including uveal melanoma, clear-cell renal cell carcinoma, gastric adenocarcinoma, colorectal cancer, and non-small-cell lung cancer, also documented a significant decrease in tumor BAP1 expression^35–39^. Notably, low expression of BAP1 significantly correlated with larger tumor size, presence of lymphatic metastasis, and advanced TNM stage, which are all predictors of poor prognosis of ICC after curative surgery^5,7^. More importantly, our results showed that ICC patients with low BAP1 expression in general had worse prognosis than those with high BAP1 expression. The findings are in accordance with previous reports that reduced expression or inactivation of BAP1 in tumors, which often results from germline-inactivating or somatic-inactivating BAP1 mutations or deletions, increases tumor susceptibility, or predicts worse clinical outcomes^13,22–27,29^. Hence, we believe that BAP1 is a candidate biomarker for prognostic prediction as well as a novel therapeutic target for ICC patients.

Our in vitro experiments showed that ectopic expression of wild-type but not inactive mutant BAP1 dramatically suppressed, while knockdown of BAP1 promoted, cell proliferation, migration, and invasion abilities in ICC cell lines. These results were highly consistent with other previous studies, in which growth-inhibiting and invasion-suppressing effects of BAP1 were reported^13,40^. Further, cell cycle analysis revealed that upregulation of wild-type but not inactive mutant BAP1 evidently reduced cells in the S phase, while arresting cells in the G0/G1 phase. Similarly, downregulation of BAP1 markedly increased cells in the S phase and reduced cells in the G0/G1 phase, consistent with the results of a recent study that depletion of BAP1 resulted in a modest accumulation of host cell factor 1, which promoted the transition from G1 to S phase^41^. Furthermore, our in vivo data confirmed that overexpression of wild-type but not inactive mutant BAP1 significantly inhibited, while knockdown of BAP1 promoted, ICC tumorigenicity, and progression. These in vitro and in vivo functional results thereby highlighted the tumor-suppressive role of BAP1 in ICC.

The MAPK protein family coordinately regulates a wide array of physiological and pathological processes, and aberrant MAPK signaling plays a crucial role in the development and progression of cancer, as well as in determining responses to cancer treatment^42^. As important members of MAPKs, hyperactivity of ERK1/2 and JNK/c-Jun signaling has been observed in a high percentage of cancers, including ICC^32,33^. Moreover, accumulating data have revealed that UCHs exert their functions in tumor onset and progression via regulating MAPK signaling pathways^34^. Hence, we hypothesized that BAP1 exerted its biological functions in ICC through modulating ERK1/2 and JNK/c-Jun signaling. As expected, we indeed found that phosphorylation of ERK1/2 and JNK/c-Jun was inhibited by wild-type but not mutant BAP1 in ICC, as shown by western blotting and immunohistochemistry. Moreover, the immunohistochemical results in ICC patients demonstrated a significantly negative correlation of BAP1 with p-ERK1/2, p-JNK, and p-c-Jun, which further confirmed these findings. ERK1/2 is activated by phosphorylation of threonine 202/tyrosine 204 and threonine 185/tyrosine 187, whereas c-Jun is activated by phosphorylation of serines 63 and 73. Phosphorylation of ERK1/2 and c-Jun induces activation of many transcription factors, such as ETS, NF-κB, and AP-1, resulting in the induction of downstream signal molecules, c-Myc, cyclin D1, and c-Fos, which are important cell-proliferating and growth-regulating factors^42^. Aberrant activation of ERK1/2, c-Jun, or their downstream targets has been reported to act as proto-oncogenes mediating cell proliferation and cell cycle progression, as well as increasing cancer cell migratory and invasion capacities^42,43^. Furthermore, we also demonstrated that hyperactivity of ERK1/2 and JNK/c-Jun signaling induced by BAP1 downregulation was necessary for ICC cell proliferation, cell cycle progression, and invasion in vitro and in vivo using the inhibitors of the ERK1/2 pathway (U0126) and JNK/c-Jun pathway (SP600125). Taken together, our data suggested that BAP1 negatively regulated the ERK1/2 and JNK/c-Jun signaling pathways to exert its tumor-suppressive functions in ICC. However, the detailed mechanisms of the upstream of MAPK cascade regulated by BAP1 in ICC need further exploration.

In summary, our study demonstrated that BAP1, which is frequently downregulated in ICC, was a putative tumor suppressor in human ICC. The tumor-suppressive effects of BAP1 proceeded by antagonizing the activity of the ERK1/2 and JNK/c-Jun signaling pathways. Importantly, downregulation of BAP1 promoted aggressive behavior of ICC, and BAP1 also served as a novel prognostic indicator for ICC patients after curative surgery. The clinical relevance and functional significance of BAP1 in ICC support its exploration as a promising therapeutic target for ICC.

## Materials and methods

### Patients and specimens

Human liver tissues were obtained from specimens of ICC patients undergoing curative surgery in the Department of Liver Surgery, Zhongshan Hospital, Fudan University. A total of 60 pairs of snap-frozen ICC samples and matched adjacent non-tumor liver tissues were collected for detecting mRNA expression of BAP1, and 12 pairs were obtained for detecting protein expression of BAP1. Another 12 snap-frozen ICC samples from patients with or without lymph node metastasis were randomly retrieved for the analysis of BAP1 mRNA and protein expression.

Archived paraffin-embedded tumor tissues from 214 consecutive ICC patients treated with curative resection between February 2001 and December 2006 were used for TMA construction and immunohistochemistry. The entrance criteria, clinical data collection, and postoperative follow-up procedures were conducted according to uniform guidelines described in our previous study^44^. Histopathological diagnosis was based on World Health Organization criteria. Tumor stage was determined in accordance with the TNM classification system established by the 2010 International Union Against Cancer^45^. OS was defined as the interval between the date of surgery and the date of death. RFS was defined as the interval between the date of surgery and the date of tumor relapse^44^.

The study was approved by the research ethics committee of Zhongshan Hospital, and informed consent was obtained from each patient according to the institutional review board protocols.

### TMA construction and immunohistochemistry

TMAs were constructed as described in our previous study^44^. Immunohistochemical staining was performed using the avidin–biotin–peroxidase complex method as described previously^44^. The primary antibodies and dilutions used were listed in Supplementary Table S1. Immunostaining intensities of these markers were semiquantitatively scored as follows: 0, negative; 1, weak; 2, moderate; 3, strong. The score of immunostaining intensity was assessed by two pathologists independently, and comparisons were performed between tumor/normal pairs. In the survival analyses, scores 0 and 1 were defined as low expression, whereas scores 2 and 3 were defined as high expression^45,46^.

### Cell lines and transfection

Two human ICC cell lines, HCCC9810 (Chinese Academy of Sciences Shanghai Branch Cell Bank, Shanghai, China) and RBE (Cell Resource Center of Tohoku University, Tohoku, Japan), were used in this study. The cell lines were cultured in RPMI-1640 medium (Gibco, Grand Island, NY, USA) supplemented with 10% fetal bovine serum (Gibco, Grand Island, NY, USA) and penicillin/streptomycin. Lentiviral vector encoding wild-type BAP1 for overexpression of BAP1 was transfected into HCCC9810 cells and designated as HCCC9810-BAP1 cells. HCCC9810-Mock cells, which were transfected with lentiviral vector alone, were used as control. Lentiviral vector encoding shBAP1 for downregulation of BAP1 was transfected into RBE cells and designated as RBE-shBAP1 cells. The target sequence of shBAP1 was listed in Supplementary Table S2. RBE-Mock cells, which were transfected with lentiviral vector alone, were used as control. After a series of preliminary studies, dose and time of U0126 (Selleckchem, Houston, TX, USA) on RBE-shBAP1 cells were selected as 10 μM and 1 h, respectively; dose and time of SP600125 (Selleckchem, Houston, TX, USA) on RBE-shBAP1 cells were selected as 20 μM and 1 h, respectively. Catalytically inactive mutant BAP1C91A was cloned into pcDNA3.1 vector and transfected into HCCC9810 cells using Lipofectamine 2000 (Invitrogen, Carlsbad, CA, USA) reagent following the manufacturer’s instructions for the in vitro study^47^. Moreover, lentiviral vector encoding inactive mutant BAP1C91A for overexpression of BAP1C91A was transfected into HCCC9810 cells for the in vivo study. Stably transfected clones were validated by qRT-PCR and western blot analysis.

### Cell proliferation, cell cycle, scratching, Matrigel invasion, western blot, immunofluorescence, and qRT-PCR assays

Cell proliferation, cell cycle, scratching, and Matrigel invasion assays were performed as described previously^44^. Protein from ICC cells or snap-frozen specimens were extracted using RIPA buffer, and protein expression was analyzed by western blot as described previously^44^. Immunofluorescence assay were also performed as previously described^45^. The primary antibodies and dilutions used were listed in Supplementary Table S1. Total RNA was extracted using Trizol reagent (Invitrogen, Carlsbad, CA, USA) and reverse transcribed to cDNA using PrimeScript RT Reagent Kit (Takara, Japan). SYBR Premix Ex Taq^TM^ (Takara, Japan) was used for qRT-PCR following the manufacturer’s instructions, and gene amplification and detection were performed using the ABI PRISM 7900 Sequence Detection System (Applied Biosystems, Foster City, CA, USA). Gene-specific primers were designed as follows: BAP1 forward, 5′-GACCCAGGCCTCTTCACC-3′; BAP1 reverse, 5′-AGTCCTTCATGCGACTCAGG-3′; GAPDH forward, 5′-AGCCACATCGCTCAGACAC-3′; GAPDH reverse, 5′-GAATTTGCCATGGGTGGA-3′.

### **In vivoassays**

Male non-obese diabetic-severe combined immunodeficiency (NOD/SCID) mice (4 weeks old) were purchased from the Shanghai Institute of Material Medicine of the Chinese Academy of Science and raised under specific pathogen-free conditions. Animal care and experimental protocols were conducted in accordance with guidelines established by the Shanghai Medical Experimental Animal Care Commission. Ethical approval was obtained from the research ethics committee of Zhongshan Hospital. HCCC9810-BAP1, HCCC9810-Mock, HCCC9810-BAP1C91A, RBE-shBAP1, and RBE-Mock cells (5 × 10^6^) were suspended in 200 μL serum-free RPMI-1640 and Matrigel (BD Biosciences; 1:1), and then injected subcutaneously into the flanks of NOD/SCID mice. Inhibition of ERK1/2 or JNK/c-Jun signaling pathways was performed by intraperitoneal injections of 25 μM/kg U0126 or 50 μM/kg SP600125 thrice a week from day 15 after inoculation, respectively. Inhibition of ERK1/2 or JNK/c-Jun signaling pathways was confirmed at the end of study using immunohistochemistry. The tumor volume was measured every 3 days with a caliper and calculated in mm^3^ using the following formula: *V* = length × width^2^/2. Upon euthanizing the mice, the tumors were removed, weighed, and photographed.

### Statistical analysis

Statistical analyses were performed using SPSS version 18.0 for Windows. Categorical data were analyzed using the *χ*^2^ test or Fisher's exact test and quantitative data were analyzed using the Student’s *t* test or one-way analysis of variance, when appropriate. OS and RFS curves were plotted using the Kaplan–Meier method and compared using the log-rank test. Univariate and multivariate analyses were performed using the Cox proportional hazards regression model. Two-tailed *P* values < 0.05 were considered statistically significant.

## Electronic supplementary material

Supplementary table 1 and table 2

Supplementary Figure S1

Supplementary figure legends
