# Supplementary material for: BAP1 acts as a tumor suppressor in intrahepatic cholangiocarcinoma by modulating the ERK1/2 and JNK/c-Jun pathways
Source: Cell Death Dis. 2018 Oct 10;9(10):1036. doi: 10.1038/s41419-018-1087-7 (PMC6179995; doi:10.1038/s41419-018-1087-7)
Supplement: Supplementary file 1 — Supplementary table 1 and table 2 [file 41419_2018_1087_MOESM1_ESM.docx]

| **Supplementary Table S1. Primary Antibodies for WB, IHC and IF.** | | | | | |
| --- | --- | --- | --- | --- | --- |
| **Antibody** | **Concentration** | **Concentration** | **Concentration** | **Specificity** | **Company** |
|  | **for WB** | **for IHC** | **for IF** |  |  |
| BAP1 | / | 1:100 | 1:50 | Mouse | Santa cruz |
| BAP1 | 1:1000 | / | / | Rabbit | CST |
| GAPDH | 1:100 | / | / | Mouse | Santa cruz |
| ERK1/2 | 1:1000 | / | / | Rabbit | CST |
| p-ERK1/2^Thr202/Tyr204^ | 1:1000 | / | / | Rabbit | CST |
| JNK | 1:1000 | / | / | Rabbit | CST |
| p-JNK^Thr183/Tyr185^ | 1:1000 | / | / | Rabbit | CST |
| c-Jun | 1:1000 | / | / | Rabbit | CST |
| p-c-Jun^Ser73^ | 1:1000 | / | / | Rabbit | CST |
| Abbreviations: WB, western blot; IHC, immunohistochemistry; IF, immunofluorescence; Santa cruz, Santa Cruz Biotechnology; CST, Cell Signaling Technology. | | | | | |

| **Supplementary Table S2. Target Sequence of shBAP1.** | | |
| --- | --- | --- |
| **Gene** | **List** | **Target sequence** |
| BAP1 | sh1 | ACAACTACGATGAGTTCAT |
|  | sh2 | TGGAAGATTTCGGTGTCAA |
|  | sh3 | TCCGTGATTGATGATGATA |
