# Supplementary figures and images for: BAP1 acts as a tumor suppressor in intrahepatic cholangiocarcinoma by modulating the ERK1/2 and JNK/c-Jun pathways
Source: Cell Death Dis. 2018 Oct 10;9(10):1036. doi: 10.1038/s41419-018-1087-7 (PMC6179995; doi:10.1038/s41419-018-1087-7)

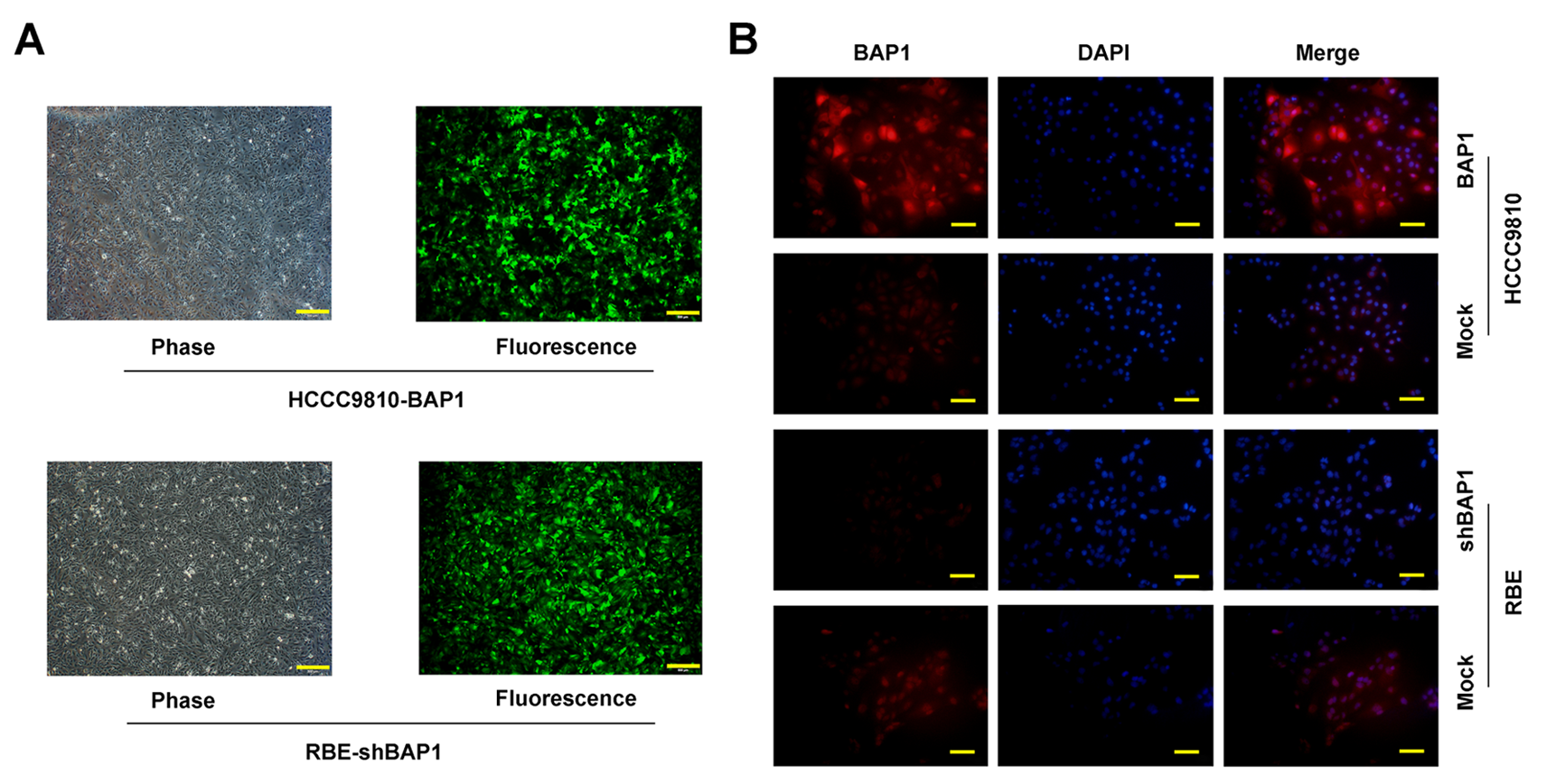

Supplement: Supplementary file 2 — Supplementary Figure S1 [file 41419_2018_1087_MOESM2_ESM.tif]
