# Supplementary material for: BAP1 acts as a tumor suppressor in intrahepatic cholangiocarcinoma by modulating the ERK1/2 and JNK/c-Jun pathways
Source: Cell Death Dis. 2018 Oct 10;9(10):1036. doi: 10.1038/s41419-018-1087-7 (PMC6179995; doi:10.1038/s41419-018-1087-7)
Supplement: Supplementary file 3 — Supplementary figure legends [file 41419_2018_1087_MOESM3_ESM.doc]

**Supplementary Figure S1.** The transfection efficiency of HCCC9810-BAP1 and RBE-shBAP1 were evaluated by immunofluorescent staining. (A) EGFP in the lentiviral vector. Scale bars = 500 μm. (B) Using specific BAP1 antibody. Scale bars = 200 μm.
